# A method for generating virus-free cassava plants to combat viral disease epidemics in Africa^☆^

**DOI:** 10.1016/j.pmpp.2018.09.002

**Published:** 2019-01

**Authors:** M.N. Maruthi, E. Charles Whitfield, Gerald Otti, Silver Tumwegamire, Edward Kanju, James P. Legg, Geoffrey Mkamilo, Robert Kawuki, Ibrahim Benesi, Anabela Zacarias, Therezia Munga, Francis Mwatuni, Edward Mbugua

**Affiliations:** a Natural Resources Institute (NRI), University of Greenwich, Chatham Maritime, Kent, United Kingdom; b International Institute of Agriculture (IITA), P.O Box 34441, Dar es Salaam, Tanzania; c Department of Agricultural Research and Development (DRD), P.O Box 2066, Dar es Salaam, Tanzania; d National Agricultural Research Organization (NARO), P.O Box 295, Entebbe, Uganda; e Department for Agricultural Research Services (DARS), Chitedze Research Station, P. O. Box 158, Lilongwe, Malawi; f National Institute of Agricultural Research (IIAM), P.O Box 3658, Maputo, Mozambique; g Kenya Agricultural and Livestock Research Organization (KALRO), Kenya; h Kenya Plant Inspectorate Services (KEPHIS), Plant Quarantine and Biosecurity Station, Muguga, P.O. Box 49592 00100, Nairobi, Kenya; i Genetic Technologies International Limited (GTIL), P.O. Box 47430-00100, Nairobi, Kenya

**Keywords:** *Manihot esculenta*, Cassava brown streak disease, Cassava mosaic disease, Virus indexing, Virus diagnosis, ACMV, *African cassava mosaic virus*, CBSD, Cassava brown streak disease, CBSV, *Cassava brown streak virus*, CMD, Cassava mosaic disease, CTAB, Cetyl trimethylammonium bromide, EACMV, *East African cassava mosaic virus*, EACMV-Ug, *East African cassava mosaic virus*-*Uganda*, LFC, Laminar flow cabinet, MS, Murashige and Skoog, PPM, Plant Preservation Mixture, RH, Relative humidity, SDW, Sterilised deionised water, UCBSV, *Ugandan cassava brown streak virus*, UIC, Unique identifying code

## Abstract

Here, we report a method to clean cassava plants from viral infections that cause cassava mosaic and brown streak diseases in Africa. Infected plants of resistant or tolerant varieties from Malawi, Mozambique, Kenya, Tanzania and Uganda were cleaned in the UK using a combination of tissue culture, chemotherapy and thermotherapy. In the first cycle of our virus-indexing procedure, we successfully cleaned 27 of the 31 varieties (87%), and after an additional three cleaning cycles, all plants were virus-free. Virus-free tissue-cultured plants were shipped back to Africa for distribution to farmers. This first cross-boundary effort provides important lessons for mitigating the two-major cassava viral diseases.

## Highlights

• We developed a method for successfully cleaning ‘best bet’ virus-resistant/tolerant cassava varieties from infections of viruses causing two devastating diseases, cassava mosaic disease and cassava brown streak disease.
• This was a multi-country major collaborative initiative in eastern and southern Africa for combating the two major viral diseases of cassava.
• This work has allowed the exchange of best cassava germplasm between the worst affected countries for rapid control of viral disease epidemics.
• Lessons learned here will help control the two most important diseases of cassava at the regional level.
• Virus-cleaned cassava lines are deposited into the International Institute of Tropical Agriculture-Nigeria germplasm collection centre.
• The cassava lines will be available as a permanent source of germplasm for future disease-resistant breeding programs.

## 1 Introduction

Cassava (*Manihot esculenta* Crantz; *Euphorbiaceae*) is a staple crop for more than 800 million people in the tropics [1]. The crop is affected by more than 100 insect and mite species and about 30 cassava diseases induced by viruses, phytoplasmas, bacteria or fungi [2]. Among the diseases, cassava mosaic disease (CMD) and cassava brown streak disease (CBSD) are the most important viral diseases in Africa [[3], [4], [5], [6]]. Epidemics of both diseases have occurred during the last two decades (sometimes simultaneously), severely affecting cassava production and threatening the livelihoods of farmers and food security in eastern African countries [7].

**Fig. 1:**
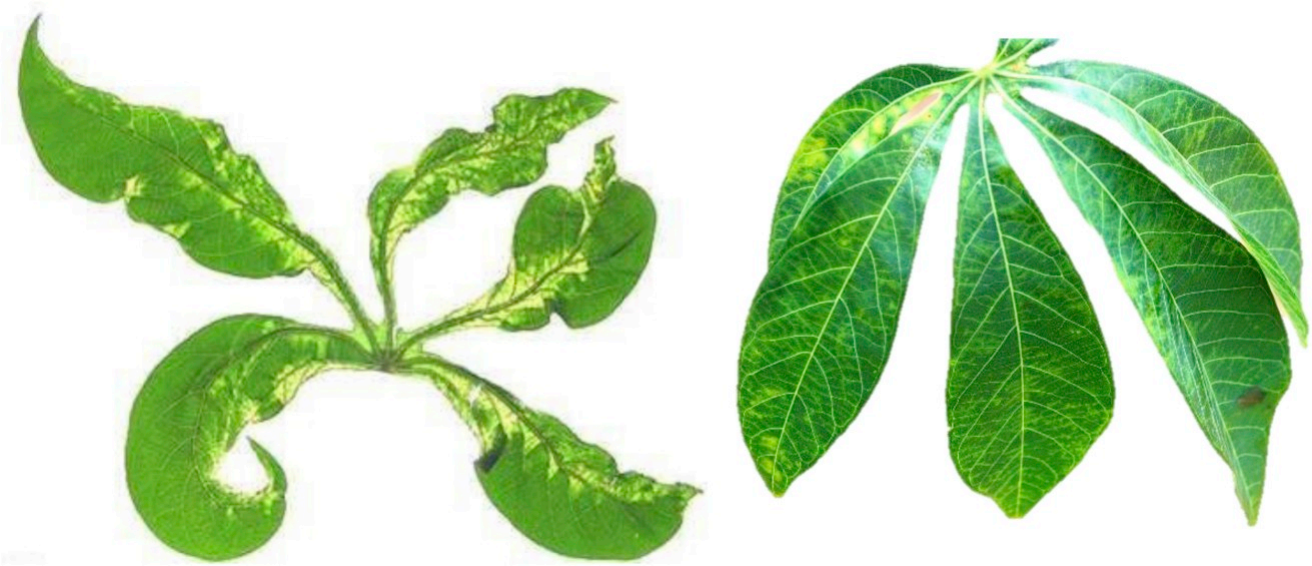
Typical symptoms of CMD (left) and CBSD (right) on cassava leaves.

CMD is caused by 11 species of single-stranded DNA viruses belonging to the family *Geminiviridae*, genus *Begomovirus*, which are collectively called cassava mosaic begomoviruses or CMBs [4,5]. These viruses are transmitted by whitefly, *Bemisia tabaci* Gennadius (family Aleyrodidae, order Hemiptera), as well as disseminated through the propagation of infected cuttings [6]. The most obvious symptoms of CMD include a characteristic mosaic of pale green to yellow chlorotic areas on leaves, usually accompanied by distortion (Fig. 1). Growth of susceptible plants infected at the cutting stage is severely stunted and there is poor development of the tuberous roots, which ultimately results in low yields [8]. The spread of an unusually severe form of CMD, the so-called “CMD pandemic”, was first recorded in Uganda in the late 1980s and subsequently spread to affect an area greater than 4 million km^2^ across 11 countries of East and Central Africa [5,6,8,9]. Since then, considerable success has been achieved in mitigating the effects of the CMD pandemic through the multiplication and dissemination of CMD-resistant varieties [5]. However, severe CMD continues to spread into newer areas. Previous estimates of CMD incidences in Africa range from 50% to 60% with estimated annual losses of US $1.2–2.4 billion [8,9].

CBSD is caused by two species of RNA viruses; *Cassava brown streak virus* (CBSV) and *Ugandan cassava brown streak virus* (UCBSV) both of which belong to the family *Potyviridae* of the genus *Ipomovirus* [[10], [11], [12]], and are collectively referred to as cassava brown streak ipomoviruses, or CBSIs. Like CMBs, CBSIs are also transmitted by *B. tabaci*, and spread by propagating infected cuttings in the field [13,14]. The prominent symptoms of CBSD appear on leaves with varying patterns of leaf chlorosis that appears in a feathery pattern along the margins of secondary and tertiary veins, which may coalesce to form chlorotic blotches (Fig. 1) [3,6,15]. There is considerable variation in the expression of foliar symptoms depending on the cassava variety, the growing conditions (temperature, rainfall and altitude), the age of the plant, and the viral species. Some cultivars show marked foliar symptoms but lack root symptoms or have a delay in the expression of root symptoms (e.g. in ‘Kiroba’), whereas other cultivars lack foliar symptoms but have affected roots [3,16].

For the last 80 years, CBSD has been endemic in low altitude areas along the Indian Ocean coast of eastern Africa, from the north-eastern border of Kenya across the Tanzanian border down as far as the Zambezi River in Mozambique, and it has been widespread along the shores of Lake Malawi [3,6,12]. More recently, CBSD was reported in Uganda, a high altitude area inland from the east African coast, and subsequently in the neighbouring countries in the Great Lakes region of East and Central Africa, including Burundi, Rwanda, southern Sudan and the eastern part of Democratic Republic of Congo [3,4,15]. The spread into the Great Lakes region is a major concern because CBSD incidences of up to 100% were recorded and has resulted in rotting of tuberous roots in virus-sensitive varieties, reducing both the quality and quantity of tuberous roots available for consumption [3,5]. Recent estimates indicate that CBSD causes great economic losses of up to US $726 million annually to African farmers [17]. The disease has been the most important cause of food insecurity in the coastal and lake zone areas of eastern Africa and is considered a serious threat to the entire cassava-growing belt of Sub-Saharan Africa [7].

CMD has been managed in many pandemic-affected countries through developing and disseminating resistant varieties, however, progress managing CBSD has been slower [5,7]. Only a small number of cassava varieties expressing a range of resistance levels are currently available in CBSD-affected countries, but even these can be infected with CBSIs [16,18,19]. Several technologies are being recommended for controlling CBSD in farmer's fields including rigorous phytosanitation and treating cassava stems with insecticides to prevent early virus infections by the vector whiteflies. However, using resistant varieties represents the best solution currently available for CBSD control. Recent findings in our knowledge about CBSD epidemiology, however, have provided new opportunities for control [14]. We have recently shown that whiteflies transmit CBSIs poorly and that an isolation distance of about 100 m is sufficient to significantly minimise the spread of CBSIs between infected and disease-free plots [14]. These results provide an indication that phytosanitary measures, such as those involving the use of virus-free planting material coupled with isolation from surrounding potential sources of infection, offer excellent potential for CBSD control. This new knowledge also highlights the value in establishing and maintaining virus-free stocks of planting material and using such stocks as the foundation for lower tiers of seed multiplication [20].

**Fig. 2:**
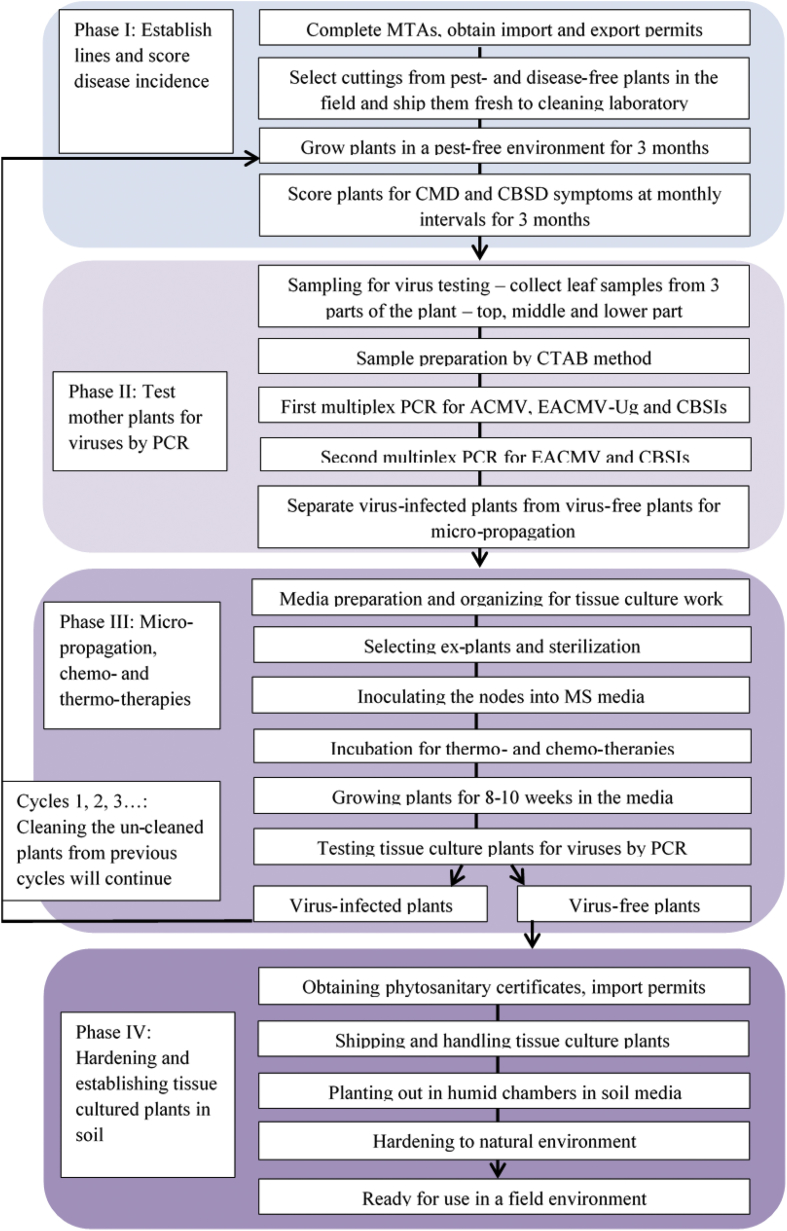
Schematic diagram of one cycle of cleaning and indexing to generate virus-free cassava plants. Each cycle is divided into four phases.

Using this new knowledge, we developed virus-indexing, tissue-culture and chemo- and thermo-therapy protocols for cleaning cassava varieties from virus infections from the five most affected countries of eastern and southern Africa (Fig. 2). These protocols have long been used for generating virus-free plants in several other crop plants including the main root and tuber crops of Africa such as cassava, yams and sweetpotatoes. In a recent study, up to 73% of the yam plants were confirmed to be free from *Yam mosaic virus* following tissue culture and heat therapies, and while the results for other viruses were inconsistent [21]. Such protocols are also adapted by the regulatory bodies in Ethiopia and Tanzania for generating certified seeds of sweetpotatoes [22]. In Africa and elsewhere, virus-free planting materials have been developed for several other crops including banana, citrus and cassava for providing healthy planting material to farmers for increased yields [20,23,24]. In this paper, we describe the use of such virus-indexing protocols at a regional level for cleaning cassava from five eastern and southern African countries of Kenya, Tanzania, Uganda, Malawi and Mozambique in the project “Cassava varieties and Clean seed to Combat CBSD and CMD project (5CP)” for providing virus-free planting material to the affected farmers.

## 2 Materials and methods

The virus cleaning was done in the UK, which was used as a ‘neutral’ location for importing virus-infected cassava plants from Kenya, Malawi, Mozambique, Tanzania and Uganda. For operational reasons, the process of cleaning cassava from virus infections and their subsequent returning back to the African institutions was divided into four key phases, which formed one “cycle” of the virus-indexing procedure (Fig. 2). Each cycle contained the following four phases:

Phase I: Establishing cassava lines in quarantine glasshouses and scoring for disease incidences.

Phase II: Testing mother plants for viruses by polymerase chain reaction (PCR).

Phase III: Micro-propagation, chemo- and thermo-therapies.

Phase IV: Shipping and hardening tissue-cultured virus-free plants.

### 2.1 Phase I: establishing cassava lines and scoring for disease incidences

Between 10 and 24 stem cuttings each of five to seven cassava varieties were collected from farmer's fields or research trials from each of five countries (Kenya, Malawi, Mozambique, Tanzania and Uganda) and shipped to the Natural Resources Institute (NRI) in the UK in July–August 2012 (Table 1). These cassava varieties represented the varieties that were most promising for resistance to CMD, CBSD or both diseases in those countries at that time. The cassava varieties were classified into resistant, tolerant or susceptible to the diseases based on their reaction to each disease as described previously [5,9,16,18]. These also include some farmer-preferred varieties and susceptible controls for comparison purposes. Upon arrival in the UK, the cuttings (each 12–15 cm in length) of all 31 genotypes were treated with a solution of the systemic insecticide Intercept (ICL Ipswich, UK) (a.i. imidacloprid 0.5 g per litre of water) for 30 min to kill scales, mealybugs, mites and any other pests prior to planting in a mixture of soil and compost (John Innes No. 2; Fargro Ltd., Arundel, UK) in small plastic pots (15 cm diameter). Each plant was labelled with a unique identification number and grown in an insect-free quarantine glasshouse at 25 ± 5 °C, 50%–60% relative humidity (RH) and L14:D10 (light:dark) hours for three months. Plants showing CMD and CBSD symptoms were recorded (Fig. 1) every week and immediately separated from the non-symptomatic plants. Non-symptomatic plants were confirmed to be virus-free by PCR in Phase II of the protocol. Symptomatic plants were either discarded or kept for cleaning from virus infections in a future cycle of cleaning.

**Table 1:** The 31 cassava varieties obtained from five eastern and southern African countries and cleaned of viral infections in the UK.

Country of origin	Variety name	Reaction to diseases		No. of stems imported and planted in the UK
		CMD	CBSD	
Kenya	F10-30-R2	Tolerant	Tolerant	20
	F19-NL	Tolerant	Tolerant	23
	LMI/2008/363	Tolerant	Tolerant	22
	Kibandameno	Susceptible	Susceptible	20
	Shibe	Tolerant	Tolerant	21
	Tajirika	Tolerant	Tolerant	20
Malawi	CH05/203	Tolerant	Tolerant	24
	Kalawe	Tolerant	Tolerant	20
	Mbundumali	Susceptible	Susceptible	21
	Sangoja	Tolerant	Tolerant	20
	Sauti	Tolerant	Tolerant	22
	Yizaso	Tolerant	Tolerant	20
Mozambique	Coliacanana	Susceptible	Tolerant	21
	Eyope	Tolerant	Tolerant	22
	Nziva	Susceptible	Tolerant	22
	Okhumelela	Tolerant	Tolerant	21
	Orera	Susceptible	Tolerant	22
Tanzania	KBH2002/066	Tolerant	Tolerant	21
	KBH2006/026	Tolerant	Tolerant	21
	Albert	Resistant	Susceptible	Previous collection
	Kiroba	Susceptible	Tolerant	Previous collection
	Kizimbani	Tolerant	Tolerant	21
	Mkombozi	Resistant	Susceptible	17
	Mkumba	Susceptible	Tolerant	21
	Pwani	Tolerant	Tolerant	21
Uganda	TZ130	Resistant	Tolerant	14
	NASE1	Resistant	Tolerant	14
	NASE3	Tolerant	Tolerant	12
	NASE14	Resistant	Tolerant	13
	NASE18	Resistant	Tolerant	10
	TME204	Resistant	Susceptible	13

### 2.2 Phase II: testing mother plants for viruses by PCR

#### 2.2.1 Total nucleic acid extraction for virus-indexing

**Fig. 3:**
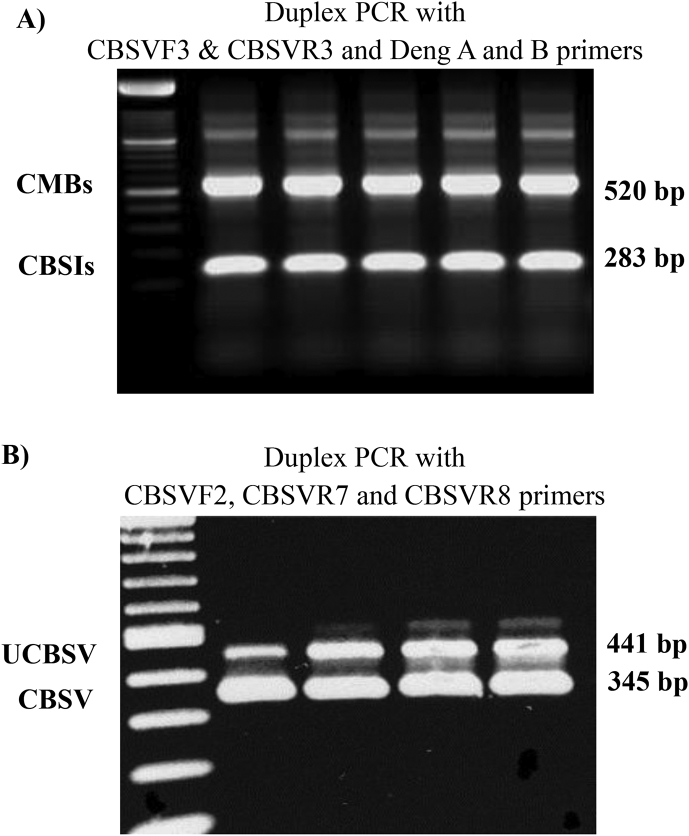
Duplex RT-PCR results (A) CMBs and CBSIs, (B) and UCBSV and CBSV. The New England Biolab's 1 Kb and 500 bp markers are used in (A) and (B), respectively. Samples amplified were mixed infections with respective viruses identified on the pictures.

Leaf samples were collected from the top, middle and bottom part of the plant and total nucleic acid was extracted from all non-symptomatic plants using a modified cetyl trimethylammonium bromide (CTAB) method [[25], [26], [27], [28]]. The optimised protocol was as follows: First, the CTAB extraction buffer (2% (w/v) CTAB, 1.4 M NaCl, 0.2% (v/v) 2-mercaptoethanol, 20 mM EDTA, 100 mM Tris-HCl, pH 8.0) was pre-heated to 60 °C for 10 min. Mercaptoethanol was added fresh to the buffer. Approximately 100 mg of fresh plant leaf tissue was placed into a thick-gauge plastic bag (10 × 15 cm) and the tissue was ground finely using a ball-bearing grinder (Qiagen Ltd. Dorset UK). Each 100 mg of ground plant tissue was then mixed with 1 mL of CTAB extraction buffer. About 750 μL of the sample was poured into a 1.5 mL centrifuge tube and heated at 60 °C for 30 min. Next, the samples were mixed with an equal volume (750 μL) of phenol:chloroform:isoamylalcohol (25:24:1) in a fume hood and centrifuged at 12,281 relative centrifugal force (g) for 10 min. Only the aqueous phase was transferred into a new 1.5 mL centrifuge tube. To precipitate the DNA, we added 300 μL of cold (−20 °C) isopropanol and incubated at −20 °C for at least 1 h. Samples were then centrifuged at 12,281 g at 4 °C for 10 min. The supernatant was discarded, and the pellet was washed with 0.5 ml of 70% ethanol, vortexed and centrifuged for 5 min at 12,281 g. Then the ethanol was removed and the pellet was vacuum-dried for 5 min. The dried pellet was suspended in 100 μL 1x TE buffer and stored at −20 °C. The extracts were diluted 1:10 fold in sterilised deionised water (SDW) before using them in PCR and reverse-transcriptase PCR (RT-PCR) for virus detection (Fig. 3).

#### 2.2.2 cDNA synthesis

Synthesis of cDNA was done using ImProm-II™ Reverse Transcriptase kit (Promega, Southampton, UK) following the manufacturer's instructions. Briefly, we prepared 5 μL Master Mix I per sample containing 1 μL each of SDW, Oligo-dT primer and 3 μL of RNA template. We incubated the mix at 70 °C for 5 min and immediately chilled on ice for 2 min. Next, we prepared 15 μL Master Mix II per sample containing 7.5 μL SDW, 4.0 μL ImProm-IITM 5X reaction buffer, 2.0 μL 25 mM MgCl2, 1.0 μL 2.5 mM dNTPs and 0.5 μL ImProm-II Reverse Transcriptase, and gently mixed in a vortexer for 3–5 s. Next, we added 15 μL Master Mix II into 5 μL Master Mix I making up a total volume of 20 μL per reaction. The reaction mixture was incubated at 25 °C for 5 min for primer annealing, at 40 °C for 60 min for cDNA synthesis, and finally at 70 °C for 15 min for inactivation of the ImPro-II™ Reverse Transcriptase. The resulting cDNA samples were stored at −20 °C until further analysis.

#### 2.2.3 Choice of PCR tests

A range of tests are available for diagnosing CMBs and CBSIs, including endpoint PCR and RT-PCR for detecting one virus at a time, as well as duplex and multiplex reactions that can detect two or more viruses in a single reaction [25,26, 29, 35]. Several real-time PCR assays are also available for CMBs and CBSIs [28,30; 34]. Tests were chosen based on the sensitivity required and the range of viruses to be tested, as well as personal preference, expertise and the facilities available. In this study, to detect CMBs (ACMV, EACMV and EACMV-Ug) and CBSIs we initially used the protocols published in Ref. [25] (Fig. 3) and subsequently [28]. A summary of the viruses detected, primers used and PCR conditions is in Table 2, Table 3.

**Table 2:** Primers used for detecting CMBs and CBSIs in uniplex, duplex and multiplex RT-PCR assays.

Primer name	Primer sequence (5′–3′)	Target^a^	Reference
**Primers used for amplifying CMBs**			
Deng A	TAATATTACCKGWKGVCCSC	Begomovirus	[31]
Deng B	TGGACYTTRCAWGGBCCTTCACA	Begomovirus	[31]
CMBRep/F	CRTCAATGACGTTGTACCA	ACMV & EACMV	[29]
CMBCP/F	GKCGAAGCGACCAGGAGAT	ACMV & EACMV-Ug	[29]
ACMVCP/R	CCCTGYCTCCTGATGATTATA	ACMV	[29]
EACMV-UG/R	CGCCTAAGCAAGGAATGGCGT	EACMV-Ug	[25]
EACMVRep/R	GGTTTGCAGAGAACTACATC	EACMV	[29]
**Primers used for amplifying CBSIs**			
**RT-PCR**			
CBSVF3	GGARCCRATGTAYAAATTTGC	CBSIs	[25]
CBSVR3	AGGAGCWGCTARWGCAAA	CBSIs	[25]
**RT-qPCR**			
CBSVR4 + CBSVF3	GCWGCTTTTATYACAAAMGC	CBSIs	[28]
**Primers used for amplifying CBSV and UCBSV individually**			
CBSVF2	GGRCCATACATYAARTGGTT	CBSIs	[25]
CBSVR7	CCCTTTGCAAARCTRAAATARC	CBSV	[25]
CBSVR8	CCATTRTCTYTCCAMADCTTC	UCBSV	[25]
**Primers used for amplifying cassava housekeeping genes**			
RubiscoLF	CTTTCCAAGGCCCGCCTCA	RubiscoL	[32]
RubiscoLR	CATCATCTTTGGTAAAATCAAGTCCA	RubiscoL	[29]
L2F	TGGTGTTGCCATGAACCCTGTAGA	Ribosomal protein (L2)	[33]
L2R	CGACCAGTCCTCCTTGCAGC	Ribosomal protein (L2)	[33]

a ACMV – *African cassava mosaic virus*; EACMV – *East African cassava mosaic virus*; EACMV-Ug – *East African cassava mosaic virus*-Uganda; CBSIs – cassava brown streak ipomoviruses; CBSV – *Cassava brown streak virus*; UCBSV – *Ugandan Cassava brown streak virus*.

**Table 3:** Primers and PCR conditions used for the efficient detection of targeted viruses.

Target virus^a^	Primer name and combinations	Primer concentration (μM)	Annealing temperature (ºC)	Expected PCR product (bp)
**Uniplex PCR**				
CBSIs	CBSVF3 CBSVR3	0.4 0.4	52	283
CBSIs	CBSVF5 CBSVR3	0.4 0.4	52	520
CBSV	CBSVF2 CBSVR7	0.4 0.4	52	345
UCBSV	CBSVF2 CBSVR8	0.4 0.4	52	441
CMBs	Deng A Deng B	0.6 0.6	52	520
ACMV	CMBRep/F ACMVRep/R	0.4 0.4	52	368
ACMV	CMBCP/F ACMVCP/R	0.4 0.4	52	650
EACMV	CMBRep/F EACMVRep/R	0.4 0.4	52	524
EACMV-UG	CMBCP/F EACMV-UG/R	0.4 0.4	52	1000
**Duplex and multiplex PCR**				
CBSIs + CMBs	CBSVF3 CBSVR3 Deng A Deng B	0.4 0.4 0.6 0.6	52	283 520
CBSV + UCBSV	CBSVF2 CBSVR7 CBSVR8	0.4 0.1 0.4	50	345 441
CBSIs + EACMV	CBSVF3 CBSVR3 CMBRepF EACMVRep/R	0.4 0.4 0.4 0.4	52	283 524
CBSIs + ACMV + EACMV-Ug	CBSVF3 CBSVR3 CMBCP/F ACMVCP/R EACMV-UG/R	0.4 0.4 0.4 0.4 0.4	52	230 650 1000
**Real-time PCR**				
CBSIs	CBSVF3 CBSVR4	0.7	68	130
Ribulose biphosphate carboxylase oxygenase gene	RubiscoLF RubiscoLR	0.7	68	171
Ribosomal protein (L2)	L2F L2R	0.7	68	135

a ACMV – *African cassava mosaic virus*; EACMV – *East African cassava mosaic virus*; EACMV-Ug – *East African cassava mosaic virus*-Uganda; CBSIs – cassava brown streak ipomoviruses; CMBs – cassava mosaic begomoviruses; CBSV – *Cassava brown streak virus*; UCBSV – *Ugandan Cassava brown streak virus*.

### 2.3 Phase III: micro-propagation, thermo- and chemotherapies

#### 2.3.1 Preparing MS medium for cassava nodal-bud culture

The protocol used to make 1 L of tissue-culture media (∼125 tubes of 8 mL each) was as follows: first, we added 2.2 g Murashige and Skoog (MS) medium to a 2 L capacity beaker, then we added 20 g sucrose and ∼950 mL of deionised water. A magnetic stirrer was used to mix the solution. Next, we added growth regulators (8 mL of NAA/L at conc. 27 μM) and Plant Preservation Mixture (PPM, Sigma-Aldrich, Dorset, UK) at 0.2–0.25 v/v % of PPM. The pH was adjusted to 5.7–5.8 using NaOH and/or HCl buffer solutions (buffer solutions should be 10% and 1% for fine adjustments). We then added deionised water to make solutions up to 1 L. Next, we added 2 g phytagel (Sigma-Aldrich, Dorset, UK) and 25 mg Ribavrin (final concentration 0.1 mM). Addition of Ribavrin was only required for media used for chemotherapy of virus-infected plant material. Each solution was mixed using a magnetic stirrer to ensure an even distribution of undissolved gelling agent while the media was pipetted into culture tubes. We added 8 mL media into clean glass tubes (e.g. Timstar, borosilicate glass, 100 × 25 mm) and sealed them with lids. The tubes were then wrapped in greaseproof paper and/or aluminium foil (minimum of two layers) and autoclaved at 121 ^°^C for 15 min. Tubes were then dried in an oven for 4 h at 60 ^°^C and finally cooled to ambient temperature before use.

#### 2.3.2 Surface sterilisation of explants

At least 20 cuttings from each of 31 genotypes were collected from the five African countries. At the time of collection, the top green parts of each plant's stem were cleaned separately, all leaves were removed and the stems were placed in plastic, labelled bags. Upon arrival in the UK, the stems (explants) were trimmed to a suitable size (usually up to 5 cm) that contained at least one nodal bud. The explants were then transferred into separate glass jars and washed 3 times in running tap water. The explants were then immersed in 70% ethanol for 3–5 s before the ethanol was poured out of the jars and replaced with a sterilisation solution (5% v/v sodium hypochlorite and 0.1 mL/L of Tween 20) that almost covered the explants. The jars were then placed on a shaker and mixed vigorously on an orbital shaker at 100 g for 20–30 min. After shaking, the explants were rinsed with SDW under aseptic conditions in a laminar flow cabinet (LFC). Explants were rinsed three to four times until no foam was left in the bottles. These sterile explants were used for seeding into tissue-culture media.

#### 2.3.3 Seeding nodal buds into MS media

**Fig. 4:**
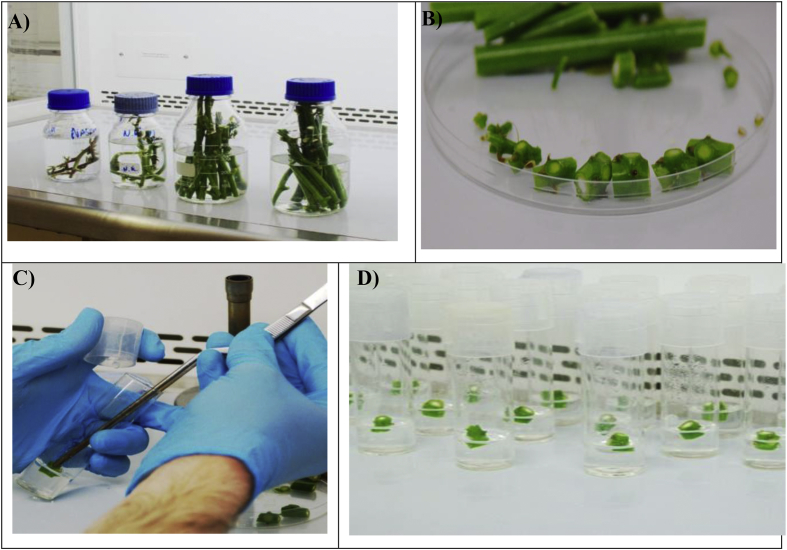
(A) Sterilisation and seeding the media with cassava nodes. Surface-sterilised cassava explants in the laminar flow ready for rinsing with SDW. (AB) Cassava node buds trimmed and ready for transferring into the culture medium. (C) Transferring nodes into the culture medium. (D). Media tubes with cassava node buds ready for labelling and incubation.

The protocol for seeding nodal buds into MS media was done inside the LFC. Prior to beginning work with the explants, the UV lamp was turned on for 10 min, the laminar flow was turned on, and the working surfaces were decontaminated with 100% ethanol. Explants were excised in sterile petri dishes using sterile knives and sterile forceps. We retained only 2–3 mm of each node bud by removing excess tissue around the bud (Fig. 4A). The node bud was then transferred into MS media using forceps ensuring that the node bud was facing upwards (Fig. 4B). The cap of the tube was closed immediately and all tubes from a single plant were labelled before seeding the next plant (Fig. 4C). Work was done near a Bunsen flame to further reduce the risk of contaminating organisms. Seeded tubes were transferred into a plant growth chamber (SLS Ltd., Wilford, UK) that was maintained at 35 °C, 50%–60% RH, and 14L:10D for heat treatment. Plants were maintained at these conditions for two weeks. Note: this step was only performed for virus-infected material receiving thermotherapy. Plants were then transferred to 25 °C growth rooms for the next 10 weeks before either being shipped back to the project's African partners or being hardened by planting into soil media for further growth in the UK.

### 2.4 Phase IV: hardening and establishing tissue-culture cassava plants in the soil

#### 2.4.1 Transplanting and hardening tissue-culture plants

**Fig. 5:**
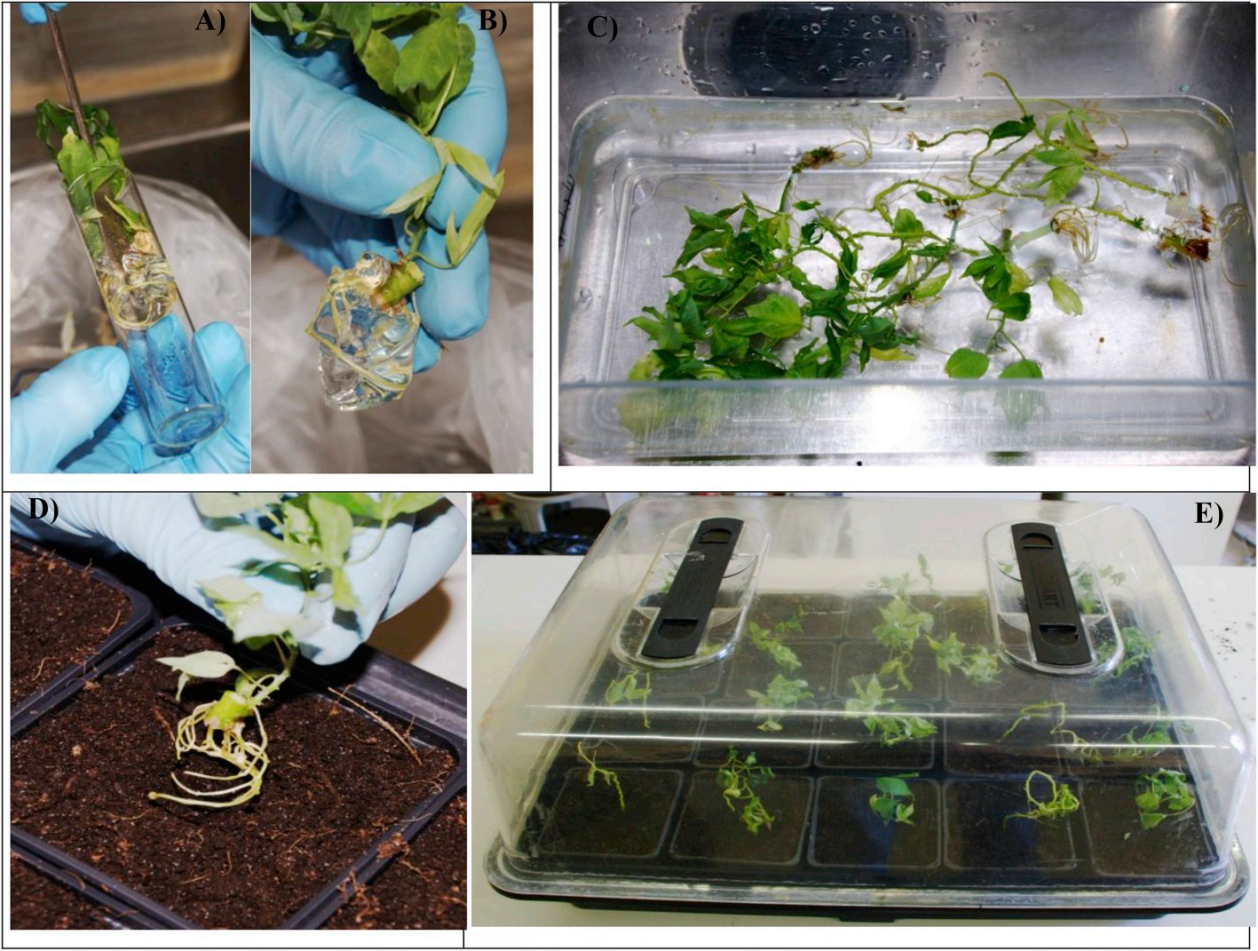
Transplanting tissue-cultured cassava plants into soil and their growth. (A) Easing the plantlet from the culture tube and (B) gently removing as much media gel as possible. (C) Rinsing any remaining gel in warm water (∼25 °C). (D) A plant being transplanted and (E) transplanted plants under propagator lid.

Plantlets were removed from the glass tubes, the media was gently washed off using tap water or SDW. The oldest and middle leaves were removed using sharp scissors, retaining only three or four developing leaves at the top of the plant. Further, to prevent fungal growth on any traces of leftover media, the plantlets were rinsed in the fungicide solution Dithane, 6 g/L (Mancozeb 80% a.i.) (ICL Ipswich, UK) for 10 min. Plantlets were placed into small pots (10 cm diameter) with compost and soil in 1:1 proportion and the pots were placed onto a propagator tray and watered just enough to soak the soil mixture (Fig. 5). After watering, the tray was immediately covered with a transparent lid that had been sprayed with water to raise the humidity. Lids were kept closed for a minimum of two weeks to allow the plants to develop new roots and leaves. Conditions were maintained at 30 ± 5 °C, 50–60% RH, 14L:10D. After two weeks, the vents in the lids were opened for one week and then we started lifting the lids incrementally using spacers between the lid and the tray. At the end of four weeks, the lids were removed completely, and we applied NPK fertiliser containing Mg (ratio of 30:10:10:2) and other trace elements (10 g dissolved in 10 L water) (Fargro Ltd., Arundel, UK). Plants were grown for an additional four weeks and then hardened by reducing the amount water provided without allowing plants to dry out completely or shed leaves. Such hardened plants can be ready to transplant into the soil media.

#### 2.4.2 Accountability and traceability of virus-cleaned plants

**Fig. 6:**
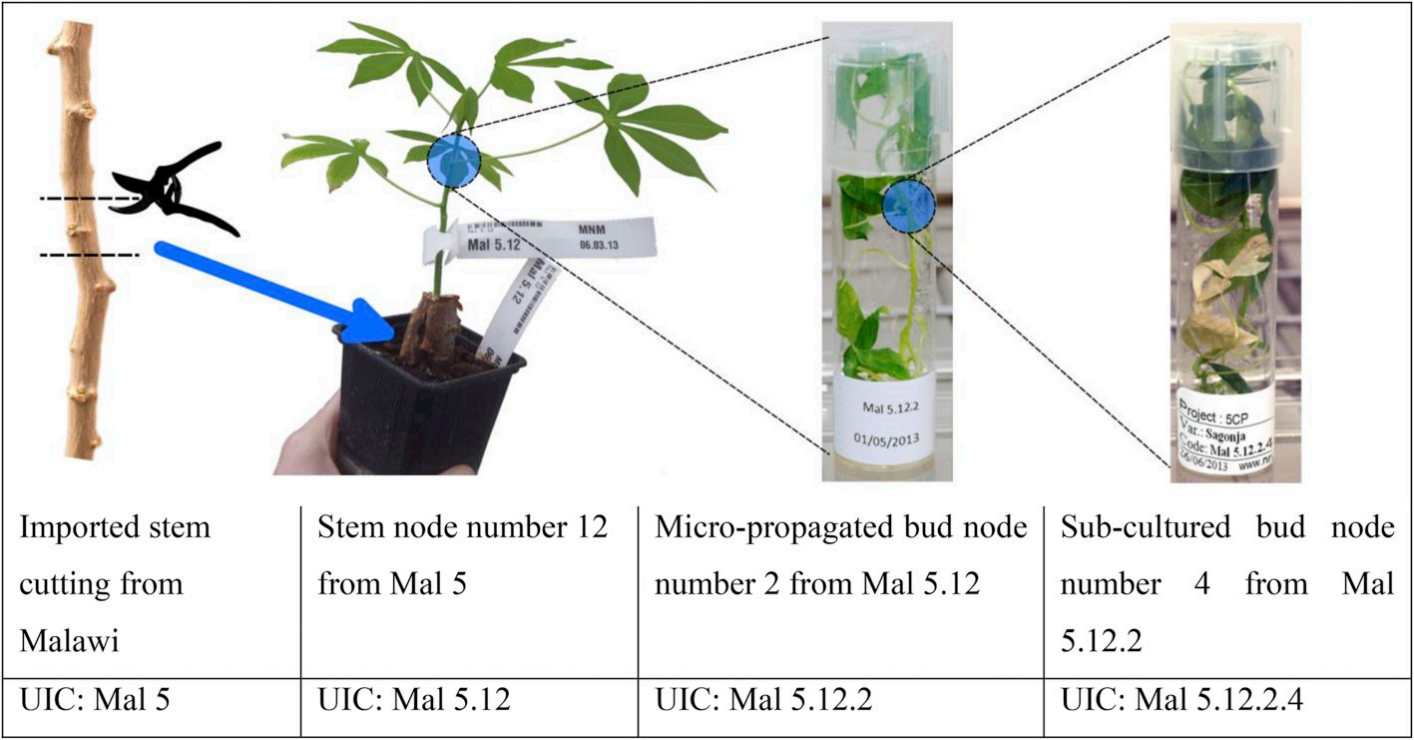
An example of the plant-labelling system using unique identification codes (UIC) at each stage of the plant multiplication process.

We designed a full traceability system that was set up to track each stem cutting from its arrival at the quarantine facility at NRI, through the micro-propagation and virus-indexing procedures, to the shipment of clean material back the partner organisations in East Africa (i.e. Phases I–IV). A well-designed traceability system that can track plant material through the processing phases to the partner organisations in Africa is essential. Such a system provides a control mechanism. If any plant material was later found to contain a virus, the traceability system could identify other potentially infected stock, thus limiting viral spread (Fig. 6).

There were two parts to the traceability system; the first was a record of all plant material. We used Microsoft Excel 2010 to keep a digital record of all the plant material in this project. A digital database is highly suitable and desirable, however a paper-based system could also be used. The second part was a labelling system that ensured all plant material, plants, and plantlets in the glasshouses and tissue-culture facilities carried a unique identifying code (UIC) (Fig. 6) that linked to the database. Each stem cutting, plant, and plantlet that was propagated had its own entry in the spreadsheet. We used a simple sequential UIC, wherein each generation of propagated or micro-propagated plants gained an additional digit. For example, for a plant showing the label “Mal 5.12.2.4” (see Fig. 6), “Mal” indicates a variety from Malawi, “5” indicates the code name given for the variety, “12” indicates a plant replicate of that variety, “2” indicates the node number on the plant (e.g. “2” would be the second node from the top of the plant, a location that is more likely to be virus-free), and “4” indicates the node number after the first sub-culturing from the previous plant. The spreadsheet was setup such that each row (entry) represented a single bud node from a plant that had been propagated, while the columns contained data on various aspects of that bud node and its history including the UIC, country of origin, cutting number, varietal common name, virus assessments (visual and PCR based), infection status, location (within glasshouse or incubator), date of tissue culture, number of nodes micro-propagated, shipping date, and destination. For each entry, additional columns were added to record details of thermo- and chemotherapies and their outcomes, number of nodes that were tissue-cultured, results of repeated PCR tests (used to confirm previous results prior to shipping), dates plantlets were hardened, dates plants were transferred to the glasshouses, or anything else pertinent to the work. The “Pivot Table” function of Excel was used to quickly generate summaries of the data and track overall progress.

We used ‘mail merge’ function to link between an Excel spreadsheet and a Word document. A template for labels was set up in a Word document, wherein each label included all the relevant fields (e.g. UIC, date, location). The ‘mail merge’ allows a linked spreadsheet to be filtered by specific fields, and then automatically generates the data into the relevant fields for each label within the Word document. We used ‘mail merge’ to automatically generate sticky labels for the micro-propagation tubes, storage and shipping containers, thus minimising the potential for human error in labelling. A label printer (LabelStation Pro 200, Advanced Labelling Ltd., Dorset, UK) and the LabelStation software were used to generate the tags and labels for plants in the glasshouse. Similar to Microsoft ‘Mail Merge’, the LabelStation software was linked to the Excel spreadsheet. Again, a label template was set up to contain the relevant fields. The link between the Excel spreadsheet and the LabelStation software allowed for data filtering, enabling us to generate plant tags for specific batches of plants with the correct UIC and other relevant information.

## 3 Results

### 3.1 Phase I: establishing cassava lines and scoring disease incidences

Between 7 and 21 stem cuttings from 31 cassava varieties were imported from Kenya, Malawi, Mozambique, Tanzania and Uganda (Table 1), which accounted for a total of 487 plants. These were grown in the NRI's quarantine glasshouse in the UK and observed for typical CMD and CBSD symptoms (Fig. 1) at monthly intervals for three months. Of these plants, 191 (39.2%) showed disease symptoms within three months and the remaining 296 were symptoms-free (Table 4). Of the 191 diseased plants, the proportion of CMD and CBSD varied; 20 were infected with CMD (10.4%) and 171 with CBSD (89.5%). The incidence of CBSD was highest for the three varieties F19-NL, Kibandameno and NASE14 (100% for all three varieties) but three other varieties, Colicanana, Orera and KBH 2002/066, were virus-free. Incidences of CMD were low on all varieties except NASE 1, which had the highest incidence (100%, Table 4). Following disease assessment, most diseased plants were destroyed when sufficient number of symptomless plants were available for PCR testing (see below). In cases where all plants were infected with one or both viruses, at least five plants were kept for subsequent virus cleaning and indexing in Phase II.

**Table 4:** CMD and CBSD incidences on cassava varieties three months after planting in the NRI glasshouse in the UK.

Country of origin	Variety	No. of CMD/total plants	% CMD incidence	No. of CBSD/total plants	% CBSD incidence
Kenya	F10-30-R2	0/13	0.0	1/13	7.7
	F19-NL	0/17	0.0	17/17	100
	LM1/2008/363	0/11	0.0	6/11	54.5
	Kibandameno	0/13	0.0	13/13	100
	Shibe	0/21	0.0	4/21	19.0
	Tajirika	0/19	0.0	2/19	10.5
Malawi	CHO5/203	1/7	14.3	2/7	28.6
	Kalawe	0/20	0.0	1/20	5.0
	Mbundumali	0/21	0.0	7/21	33.3
	Sagonja	1/19	5.3	10/19	52.6
	Sauti	1/20	5.0	2/20	10
	Yizaso	0/18	0.0	9/18	50.0
Mozambique	Colicanana	0/21	0.0	0/21	0.0
	Eyope	1/22	4.6	2/22	9.1
	Nziva	1/22	4.6	3/22	13.6
	Oekhumelela	1/21	4.8	1/21	4.8
	Orera	0/21	0.0	0/21	0.0
Tanzania	KBH 2002/066	0/17	0.0	0/17	0.0
	KBH 2006/26	1/20	5.0	16/20	80.0
	Kizimbani	0/17	0.0	7/17	41.2
	Mkumba	0/19	0.0	10/19	52.6
	Pwani	0/21	0.0	18/21	85.7
Uganda	TZ 130	0/14	0.0	9/14	64.3
	NASE1	12/12	100	3/12	25.0
	NASE3	1/12	4.8	4/12	19.0
	NASE14	0/13	0.0	13/13	100
	NASE18	0/9	0.0	4/9	44.4
	TME 204	1/10	10.0	1/10	10.0

### 3.2 Phase II: testing mother plants for viruses by PCR

We used a combination of diagnostic methods [25,28] to test plants for viral infections. A total 221 symptomless cassava plants were tested by PCR for CBSIs and CMBs. Of these, 190 (85.9%) were confirmed to be completely virus-free (Table 5). The remaining 31 (14.0%) were positive for at least one of the four viruses tested (ACMV, EACMV, EACMV-Ug and CBSIs); these plants were considered symptomless carriers. Plants showing even a faint band on gel electrophoresis of PCR products for any of the four viruses were considered infected. Virus testing by PCR and RT-PCR was therefore essential for eliminating symptomless carriers of viruses. Symptomless carriers were either discarded when sufficient clean plants were available or used in Phase III for cleaning from virus infections. In summary, one or more plants of the 27 of the 31 cassava varieties were found to be free from CMBs and CBSIs after scoring for disease symptoms in Phase I and virus testing in Phase II (Table 4). All plants of the remaining four varieties, F19-NL, Kibandameno, NASE 1 and NASE 14, were still infected with viruses. Diseased and virus-free plants were propagated separately in Phase III.

**Table 5:** A summary of the PCR testing of symptomless cassava varieties at the first cycle of the virus indexing procedures in the project 5CP.

Country of origin	Variety	No. virus-free plants/total symptomless tested (%)	No. +ve for CBSIs/total tested (%)	No. +ve for ACMV/total tested (%)	No. +ve for EACMV/total tested (%)	No. +ve for EACMV-Ug/total tested^a^
Kenya	F10-30-R2	12/12	0/12	0/12	0/12	0/12
	F19-NL	0/1	1/1	0/1	0/1	0/1
	LMI/2008/363	6/6	0/6	0/6	0/6	0/6
	Shibe	11/15 (73.3)	3/15 (20.0)	1/15 (6.7)	0/15	0/15
	Tajirika	10/14 (71.4)	4/14 (28.6)	0/14	0/14	0/14
Malawi	CHO5/203	5/6 (83.3)	0/6	1/6 (16.7)	0/6	0/6
	Kalawe	12/12	0/12	0/12	0/12	0/12
	Mbundumali	9/9	0/9	0/9	0/9	0/9
	Sagonja	8/8	0/8	0/8	0/8	0/8
	Sauti	10/11 (90.9)	1/11 (9.1)	0/11	0/11	0/11
	Yizaso	7/7	0/7	0/7	0/7	0/7
Mozambique	Colicanana	11/12 (91.6)	1/12 (8.3)	0/12	0/12	0/12
	Eyope	11/11	0/11	0/11	0/11	0/11
	Nziva	10/10	0/10	0/10	0/10	0/10
	Oekhumelela	10/10	0/10	0/10	0/10	0/10
	Orera	9/9	0/9	0/9	0/9	0/9
Tanzania	KBH 2006/26	3/3	0/3	0/3	0/3	0/3
	KBH 2002/066	9/12 (75.0)	2/12 (16.7)	1/12 (8.3)	1/12 (8.3)	1/12 (8.3)
	Kizimbani	9/14 (64.2)	5/14 (35.7)	0/14	0/14	0/14
	Mkumba	8/9 (88.8)	1/9 (11.1)	0/9	0/9	0/9
	Pwani	0/2	2/2	0/2	0/2	0/2
Uganda	TZ130	3/5 (60.0)	1/5 (20.0)	1/5 (20.0)	0/5	0/5
	NASE1	7/8 (87.5)	0/8	1/8 (12.5)	0/8	0/8
	NASE18	3/5 (60.0)	1/5 (20.0)	1/5 (20.0)	0/5	0/5
	TME 204	6/7 (85.7)	1/7 (14.3)	0/7	0/7	0/7

a ACMV – *African cassava mosaic virus*; EACMV – *East African cassava mosaic virus*; EACMV-Ug – *East African cassava mosaic virus*-Uganda; CBSIs – cassava brown streak ipomoviruses.

### 3.3 Phase III: micro-propagation, therapy treatments and repeat testing for viruses

Plants free of CMD and CBSD symptoms and confirmed to be free of viruses by PCR tests were used for further propagation and multiplication by tissue culture. About 100 node buds were planted in tissue culture media, of which 80–90 healthy plantlets with robust root and shoot growth were obtained for each of the 27 varieties. The remaining plantlets either had poor root/shoot growth or were contaminated with fungi and bacteria (usually <10%). The healthy plantlets were grown in the tissue culture media for a minimum of four weeks. A second round of virus testing was done on these plantlets and confirmed to be virus-free. Such plantlets were certified to be free from the infections of CMBs and CBSIs and shipped to the project partner GTIL in Kenya for further propagation and subsequent distribution to project partners.

Of the 31 cassava varieties, four (F19-NL, Kibandameno, NASE 1 and NASE 14) were still infected with viruses at the end of Phase II. These were propagated separately by tissue culture and subjected to thermo- and chemotherapy treatments. About 50 plantlets of each variety were grown in tissue-culture media for six weeks, and instead of shipping them to Kenya for further propagation, these plants were planted back into soil and compost at the NRI quarantine glasshouses for plant growth, surveillance for disease symptoms and subsequent virus testing. This constituted the beginning of a second cycle of virus indexing protocols. Similar to Cycle 1, plants were grown for three months and the entire process (scoring for disease symptoms, PCR testing, tissue culturing, and therapy treatments) was repeated for these varieties in the second cycle. However, results from the second cycle were disappointing as none of the plants from these varieties were virus-free at the end of Cycle 2, although the severity of CBSD symptoms was significantly lower compared to initially field-collected plants. We speculate that the lower severity in symptoms was the result of reduced viral load in the tissue-cultured plants because of thermo- and chemo-therapy treatments. A third cycle of virus indexing procedures was started on the four varieties by repeating the entire process of growing plants in the quarantine glasshouse for three months, scoring for disease symptoms, PCR testing, tissue culturing and therapy treatments. Fortunately, these plants were found to be free of CMBs and CBSIs at the end of the third cycle of cleaning, and these were certified to be virus-free and shipped to GTIL Kenya for further multiplication and distribution [20].

### 3.4 Phase IV: shipping and hardening tissue-cultured virus-free plants

Obtaining an import permit from the Kenya Plant Health Inspectorate Services (KEPHIS) and a phytosanitary certificate from the UK Department for Environment, Food and Rural Affairs (DEFRA) was a prerequisite for shipping cassava plants back to Kenya. DEFRA requires submission of a disease diagnostic report based on the conditions laid out in the KEPHIS import permit. The conditions included testing plants for infections by CMBs and CBSIs using PCR/RT-PCR and confirming them to be free of viruses, as well as cleaned of any visible contaminations. Meeting these conditions was an easy task using the protocol described herein; we had previously tested for CMBs and CBSIs twice (once on mother plants and again on tissue-cultured plants) and removed all contaminated plants from inspections. This allowed us to obtain the DEFRA phytosanitary certificate easily for shipping the plants.

For shipping, the glass tubes containing the tissue-culture plantlets were placed into plastic tube racks and packed into large sturdy cardboard boxes with sufficient padding around the racks to minimise the impact of handling during transportation. The tissue-culture media used for plantlets to be shipped had a slightly higher concentration of phytagel (2.2 g/L instead of the normal 2 g/L), which provided a sturdier medium that reduced the chances of plantlet damage during transit. Both import permit and phytosanitary certificates were included into the courier parcel to facilitate clearance at Kenya's customs department. Approximately 3000 certified cassava plantlets (about 100 for each variety) were shipped to GTIL Kenya. We communicated with the GTIL prior to shipping plants to minimise delays in customs clearance. Tissue-cultured cassava plantlets can survive in the dark conditions of a parcel pack for up to a week, but any further delays will negatively affect their survival. It is advisable to open the parcel immediately upon receiving it and put the plantlets under bright light in a tissue-culture room or expose to natural light under a shade. Plants should be allowed to acclimatise to the new conditions for about a week before opening the tubes to begin the hardening process. In our project, these steps were done seamlessly because our partner that received the plants, GTIL in Kenya, has the requisite experience working with tissue-cultured plants. GTIL multiplied these cleaned plants and about 2000 plantlets were redistributed to partners in Kenya, Malawi, Mozambique, Tanzania and Uganda [20].

## 4 Discussion

This study contributed to the first regional attempt in eastern and southern Africa to control the two major viral diseases of cassava by exchanging the best virus-resistant/tolerant varieties between the worst affected countries. The cassava varieties were sent to the UK as a ‘neutral venue’ for cleaning 31 cassava varieties from infections of CMBs and CBSIs by virus indexing, tissue culture and therapy treatments. They were cleaned successfully and shipped back to the African partners for multiplication and subsequent distribution to farmers [20]. The process of cleaning indeed started while collecting cassava stem cuttings in farmer's fields in the African countries. Care was taken to select cuttings from plants free of pest and disease symptoms to facilitate easier cultivation in the quarantine glasshouse in the UK. Scale insects, mealybugs and mites would add additional challenges during viral cleaning because these pests are difficult to control in the contained environment of a glasshouse. Whenever possible, we also selected plants free of viral symptoms because starting with plants that have low or no viral load greatly reduces the time and effort required to eliminate viruses. In our experience, susceptible plants of the four cassava varieties with severe viral infections required 2–3 cycles of therapy and tissue culturing and this took up to two years (Fig. 1). Tolerant and resistant plants were cleaned in the first cycle, which took only about 8–10 months. Cleaning of vegetatively-propagated crops like cassava in this study and bananas, potatoes, sweetpotatoes, sugarcane and other crops by others has been done before [21,22,24]. However, cleaning and germplasm exchange at regional level is the first of its kind to our knowledge for any vegetatively-propagated crop and sets an example to follow for other crops for better disease control, improved yields and food securities. Use of insecticides have been increasingly recommended for controlling the whitefly vector of CMBs and CBSIs [5,7]. Our virus-free cassava varieties that have high levels of tolerance to CMD and CBSD can therefore be part of an integrated program for controlling both whiteflies and viral diseases.

A critical part of our method for virus indexing and cleaning was careful monitoring and surveillance of each plant. Plants were grown for three months and scored for disease symptoms every month. Surveillance of each plant throughout the three-month growth period was critical because some tolerant varieties have longer incubation periods before the expression of viral symptoms. A three-month growth period thus minimises false positives (plants incorrectly identified as virus-free) and reduces the number of plants that need to be confirmed virus-free by PCR and subsequent tissue culturing in Phase II [23]. A critical examination of symptoms therefore saves both time and resources while minimising the multiplication of virus-infected plants. It is important to note, however, that although CMD symptoms are easily recognisable, scoring for CBSD was done by an experienced researcher based on detailed description of symptoms and how they are affected by environments [3,16].

The PCR tests were chosen by the sensitivity required based on the source of the sampled material. For example, the recent real-time PCR Taqman assays [28] which were up to 300% more sensitive were preferred over the end-point PCR methods [25,26] when they became available in our laboratories. We found that collecting leaf samples from three parts of the plant (top, middle and bottom) and pooling them together during sample preparation greatly increased the chance of virus detection because viruses that are restricted to one part of the plant are more likely to be detected using this sampling method. We adopted this sampling method for testing all plants in this study [28].

Another critical aspect of this work was adapting a ‘zero tolerance’ policy for virus testing to prevent inadvertent virus introduction and movement between the affected countries. To prevent this, all plants were tested twice by PCR for CMBs and CBSIs during the cleaning process. The first testing was done before tissue culturing of the mother plants and thus prevented multiplication of infected material at the very beginning of the process. The second testing was done after the tissue culturing was done and before the plants were shipped out to Africa to make sure plants were indeed virus-free. Unlike leaf collection from the mother plants in the glasshouse for virus testing, collecting representative leaf samples from the tissue-culture plants that were grown in small glass tubes (2.5 cm diameter) was difficult because of their small size. We used sharp forceps to collect 2–3 leaves from each plantlet in aseptic conditions in a laminar flow hood but without disturbing plant's root system. Leaf samples of all plantlets from a single mother plant were pooled for virus testing by real-time PCR [28]. The traceability procedures ensured that any plantlets found to contain virus at this stage could be linked back to the original mother plant and all other associated cultured plantlets derived from that same mother plant. In our project, these tests were done using real-time PCR (which has higher sensitivity) than the end-point PCR protocols [25,26] but did not detect CMBs or CBSIs from any tissue-cultured plants. This confirmed that the approach that we have used for cleaning cassava from CMBs and CBSIs has worked. Such plantlets were certified to be virus-free and shipped to the project partner Genetic Technologies International Limited (GTIL) in Kenya. The GTIL further multiplied the plants in their tissue culture facilities and distributed to project partners in Kenya, Tanzania, Malawi, Mozambique and Uganda [20].

Hardening is an important process in establishing tissue-cultured plants. Complete loss of plants can occur if proper care is not taken during the hardening process. At this stage, plantlets are highly susceptible to infections by fungi, attack by root insects, low humidity and high temperatures. Consequently, the plantlets should be kept in a clean, pest-free environment and provided with enough water to maintain humidity but not so much water that plants rot. Fertilisers should only be applied after plants are well established. The process from initial transplanting to complete hardening takes about 8–10 weeks depending on the cassava variety and specific growth conditions. Plants develop quicker in warmer temperatures (30–35 °C) and bright long days. However, temperatures exceeding 40 °C such as in poly-houses can severely hinder plant growth or result in plant death. Good care of the plants was therefore taken which resulted on average > 90% plant survival after planting into the soil.

An important but often neglected aspect of a virus cleaning and indexing system is traceability of the plants. As each plant is propagated, PCR tested, cleaned of viruses, and multiplied to hundreds of plantlets, it is essential to check for viruses, if any, manifested in subsequent steps. If an infection is discovered, any plants linked to the infected plant could then easily be re-tested, cleaned, or removed without having to discard a larger batch of plants. During this project, we employed a relatively simple but encompassing traceability system using commonly available software such as the Microsoft's Excel workbooks. This system allowed us to manage more than 5000 plants through phases I to IV and ensured that we generated and shipped only certified clean material of known origins to our project partners in eastern and southern Africa. It is important to remember that a traceability system is only as good as the data that is put into it, so it is vital that all data input into the system are accurate. Correct labelling and tagging of all material can minimise mistakes. We used the automated labelling systems to reduce human error that can occur when writing hundreds of labels by hand. Finally, we suggest that if the data in a traceability database is discovered to be incorrectly entered, all the material in that run should be destroyed, and the data should be separated from the main documents to avoid the risk of miscategorising material. In summary, we have demonstrated that it is possible to clean the vegetatively-propagated crops such as cassava from two major virus infections for germplasm exchange at the regional level. This can serve as an example for cleaning and exchange of germplasm of other vegetatively-propagated crops such as bananas, potatoes, sweetpotatoes, sugarcane and others.

## 5 Conclusions

In this multi-country collaborative project, we developed a method for successfully cleaning 31 resistant/tolerant cassava varieties from infections of CMBs and CBSIs. Plants were taken from the areas of eastern and southern Africa that are most affected by CMD and CBSD: Kenya, Malawi, Mozambique, Tanzania and Uganda, and represented the most promising virus-resistant varieties of cassava in each country. This work has allowed the exchange of best cassava germplasm between the worst affected countries and this is contributing to control the two most important diseases of cassava at the regional level. The clean cassava lines are now deposited into a permanent collection at the International Institute of Tropical Agriculture-Nigeria germplasm collection centre, thus making them available for many generations to come as sources of disease-resistant stock.

## Conflicts of interest

The authors declare no competing interests.

## Author contributions

MNM led the NRI part of the project coordinating collection and delivery of cassava germplasm from the five African countries. CW carried out virus-cleaning work and GO contributed to virus indexing by PCR testing at NRI.

ST, EK and JPL were the lead project team from IITA, developed the concept, secured the grant and contributed to collection of the germplasm.

GM, RK, IB, and AZ were country partners from Tanzania, Uganda, Malawi and Mozambique, respectively. TM, FM and EM, all from Kenya, provided leadership, quarantine advice and tissue-culture propagation, respectively.
